# Effect of Modified Alkaline Supplementation on Syngenic Melanoma Growth in CB57/BL Mice

**DOI:** 10.1371/journal.pone.0159763

**Published:** 2016-07-22

**Authors:** Tommaso Azzarito, Luana Lugini, Enrico Pierluigi Spugnini, Rossella Canese, Alessio Gugliotta, Stefano Fidanza, Stefano Fais

**Affiliations:** 1 Department of Drug Research and Medicine Evaluation, National Institute of Health, Rome, Italy; 2 SAFU Department, Regina Elena Cancer Institute, Rome, Italy; 3 Department of Cell Biology and Neurosciences, National Institute of Health, Rome, Italy; IDI, Istituto Dermopatico dell'Immacolata, ITALY

## Abstract

Tumor extracellular acidity is a hallmark of malignant cancers. Thus, in this study we evaluated the effects of the oral administration of a commercially available water alkalizer (Basenpulver®) (BP) on tumor growth in a syngenic melanoma mouse model. The alkalizer was administered daily by oral gavage starting one week after tumor implantation in CB57/BL mice. Tumors were calipered and their acidity measured by *in vivo* MRI guided ^31^P MRS. Furthermore, urine pH was monitored for potential metabolic alkalosis. BP administration significantly reduced melanoma growth in mice; the optimal dose in terms of tolerability and efficacy was 8 g/l (p< 0.05). The *in vivo* results were supported by *in vitro* experiments, wherein BP-treated human and murine melanoma cell cultures exhibited a dose-dependent inhibition of tumor cell growth. This investigation provides the first proof of concept that systemic buffering can improve tumor control by itself and that this approach may represent a new strategy in prevention and/or treatment of cancers.

## Introduction

The tumor acidic microenvironment is a highly hostile milieu that poses a challenging riddle to clinical researchers. This acidity is the result of the abnormal glucose consumption by tumor cells [1–3]. In this regard, one of the most studied biochemical anomaly in tumor cells is the Warburg effect, which is a shift from ATP generation through oxidative phosphorylation to ATP generation via glycolysis, even under normal oxygen concentrations [4,5]. Consequently, neoplastic cells frequently derive an abnormally large share of their energy from aerobic glycolysis, converting most incoming glucose to lactate rather than metabolizing it in the mitochondria through the Kreb’s cycle [4,5]. Although ATP production by glycolysis is more rapid than by oxidative phosphorylation, it provides a much lower energy gain. This handicap leads to the cancer cells greatly increasing their glucose uptake to meet their amplified energy, biosynthesis and redox requirements, resulting in lactate accumulation and the disposal of this end product through the H^+^ extrusion by proton transporters [6]. There are several anomalies originating from this peculiar metabolism: 1) reversal of the pH gradient (tumors have a basic cytoplasm and highly acidic organelles and extracellular microenvironment); 2) selection within the tumor cells of the more aggressive phenotypes that thrive in this highly challenging environment; 3) induction of a state of anergy in the regional lymphocytes that over time results in their death; 4) activation of the tissue metalloproteases that promote local invasion; 5) impairment of chemotherapy effectiveness [6–12]. Chemoresistance occurs through a protonation reaction that mostly happens in the extracellular compartment (a large majority of chemotherapy agents are weak bases) and sequestration within the acidic organelles in the tumor cytoplasm of those molecules that enter despite the pH counter-gradient (a basic molecule diffusing from an acidic compartment towards a basic one) [3,8,9].

All these conditions have resulted in the failure of most current antitumor strategies at controlling tumor growth and diffusion, especially for solid neoplasms [9]. Recent investigations from our group and other researchers pointed out that countering the tumor acidification might reverse chemoresistance and improve tumor control and overall survival [13–17]. The strategy of inhibiting specific classes of proton transporters has been tested through the off-label use of commercially available drug molecules. In particular, our group has successfully used proton pump inhibitors (PPIs) to impede tumor V-ATPase function [13–24]. This strategy resulted in the delay or arrest of tumor growth in companion animals with spontaneous neoplasms as well as in humans affected by osteosarcoma and metastatic breast carcinoma [25–27]. Furthermore, in a preliminary investigation we observed that systemic alkalization combined with PPIs and metronomic chemotherapy in pets resulted in increased efficacy compared to standard metronomic chemotherapy [28]. This study approached the problem of tumor acidity in a more thorough fashion; not only by blocking the proton pump but also by neutralizing free protons with a systemic buffer [29,30]. Likewise, from a preclinical investigation on TRAMP mice, we inferred that alkalization had a protective effect against the development of spontaneous prostatic carcinoma. In this research, sodium bicarbonate had been provided with the water to mice starting at different early ages (3,5,7 and 10 weeks). The results have been impressive since these mice that have a physiological disease penetrance of 100% at defined times for prostate carcinoma, did not develop the neoplasia. Indeed all the laboratory animals treated at a very early age (3 and 5 weeks) had complete protection from the occurrence cancer, although 70% still developed prostatic hypertrophy [31]. Concerning the hypothesis of direct buffering with sodium bicarbonate, we found this choice as suboptimal considering that the maximum obtained value of pH (8.5) is close to the pH of some mineral waters. Additional drawbacks to this strategy that might negatively affect its clinical implementation include: 1) a saturated sodium bicarbonate solution is unpalatable to humans 2) alkalization with sodium bicarbonate alone results in increased plasma sodium concentrations, which can aggravate hypertension, cardiac arrhythmia, cardiac conduction abnormalities and water retention [32–38]. On the basis of these considerations we decided to move to a more ideal mix of alkaline salts with a broad spectrum of buffering action (i.e. Basenpulver®, BP). In this study we wished to verify if 1) it could be possible to counter tumor growth by using a water alkalizer as a single agent, 2) a mix of carbonate and bicarbonate salts having a physiological concentration of cations (sodium, calcium, potassium, magnesium) could avert the metabolic complications expected with the administration of sodium bicarbonate alone, and iii) the possibility of extended efficacy of a mix of carbonate and bicarbonate due to the conversion of carbonate into bicarbonate upon the consumption of the latter. For this purpose we chose to use BP, a balanced water alkalizer, whose composition is well-characterized. The aim of this study was to assess the feasibility and usefulness of this strategy in a syngenic murine melanoma model. Melanoma has been chosen because it is one the most acid neoplasms accordingly to current literature, it is a well-known chemoresistant neoplasm, it is prone to early metastasis, and has a high mortality rate in patients undergoing late diagnosis [16,18,19,23,24].

## Materials and Methods

### Chemicals and Reagents

Basenpulver® (100g of mixture contained: calcium carbonate 38.33 g, sodium bicarbonate 30.8 g, magnesium carbonate 23.1 g, disodium phosphate 3.8 g, potassium bicarbonate 3.8 g and zinc sulfate 0.17 g) was provided by Named (Milan, Italy). Sodium bicarbonate, hydrochloric acid 37% and RPMI 1640 supplemented with L-glutamine and without sodium bicarbonate, were purchased from Sigma-Aldrich (Milan, Italy). Sevoflurane inhalation anesthetic (1,1,1,3,3,3-hexafluoro-2-(fluoromethoxy) propane) was purchased from Abbott SpA, Latina, Italy. The cell impermeant ^31^P reporter 3-APP (3-aminopropyl phosphonate) was purchase from Sigma-Aldrich (Milan, Italy). Antibiotics (DE17-603E), trypsin/EDTA (BE17-171E) and fetal bovine serum (FBS) (DE14-701F) were purchased from Lonza (Milan, Italy).

### *In vitro* study

The murine B16F10 and the human A375 melanoma cell line was purchased from ATCC (Milan, Italy); the Mel501, Me30966 and WM793 human melanoma cells were kindly provided by Fondazione IRCCS Istituto Nazionale dei Tumori, Milan, Italy. Cells were seeded in 96 wells plates at the concentration of 5000 cells/well. The cells were maintained in RPMI culture medium with 10% of FBS at pH 7.4. In order to promote the solubility of BP, culture medium was acidified with HCl 1N until a pH value of 1 was achieved, whereupon the pH was increased to 7.0 with the addition of either sodium bicarbonate or BP at 4, 8 and 16 g/l. Growth inhibition was assessed at 24 and 48 hours using the 4-nitrophenyl phosphate disodium salt hexahydrate in acid phosphatase test (Sigma-Aldrich, Milan, Italy).

### *In vivo* studies

All the studies were approved by the ethical committee of the Italian National Institute of Health (Rome) and were conducted in accordance with the current Italian Law (Law 26/2014) that regulates experiments in laboratory animals. CB57/BL mice were purchased from Harlan Laboratories (UK) and housed in the animal facility of the Italian National Institute of Health. Mice had 10 and 14 hours periods of light and darkness respectively, were housed in 5 animal cages with *ad libitum* mice chow (Mucedola, Italy) and water provided through a bottle. 5 x 10^5^ B16F10 melanoma cells were subcutaneously injected in the right flank. Tumors were calipered twice a week and mice were weighted once a week. Mice were divided into a control group and treatment groups receiving increasing doses of BP diluted in water at the concentrations of 4, 8 and 16 g/l administered by oral gavage as a single daily dose. Each group consisted of 5 animals for statistical significance. The experiment was duplicated. Urine pH (obtained by free collection) was measured by using commercially available urine strips once a week. Mice were checked twice a week by a veterinarian responsible for animal welfare monitoring for signs of sufferance such as weight loss, decreased water and food consumption, poor hair coat, decreased activity levels and tumor ulcerations. Endpoints were maximum tumor volume of 1200 mm^3^ accordingly to the guidelines for a correct laboratory practice and signs of poor quality of life as described above [39].

### *In vivo* MRI guided ^31^P MRS

A group of 12 CB57/BL mice carrying syngenic melanoma tumors B16F10 implanted in the right flank were subjected to an MRI study. Upon reaching the tumor volume of 800 mm^3^ mice were gavaged with a single non-toxic dose of 4 or 8 g/l of BP solubilized in 200 μl of water. Mice underwent MRI/MRS analyses by using a Varian Inova 200/183 MRI/MRS system for small animals operating at 4.7 T. Animals were anesthetized with sevoflurane 2.5% in O_2_ 1l/min. Throughout the anesthesia procedures the ECG, PO2,and PCO2, were routinely monitored as per ISS guidelines and current literature [40]. Temperature was maintained at 37±0.5°C by a feedback controlled water circulating heating cradle. Tumor extracellular pH (pHe) value was measured from chemical shift difference between the exogenous cell impermeant ^31^P reporter 3-APP resonance and that of α-ATP. The 3-APP probe (128 mg/kg) was administered i.p. immediately prior to MRI/MRS analyses. A three turn ^31^P surface coil specifically designed to fit superficial tumors combined with a butterfly ^1^H coil (RAPID Biomedical, Rimpar, Germany) for shimming and positioning of the volume of interest (VOI) was used. T1-weighted gradient-echo multislice contiguous images (TR/TE = 400/3.5 msec, a α 70°, thickness = 1 mm, 8 averages, 19 slices, matrix 128 x 128, FOV = 3 x 3 cm^2^ which correspond to in plane resolution of 0.2 x 0.2 mm^2^) were acquired to localize the tumor. ^1^H localized spectra were used to optimize magnetic field homogeneity in order to to increase the signal resolution within the tumor (^1^H PRESS, TR/TE = 2,000/23 msec). ^31^P localized spectra were acquired from the tumor with a pulse-acquire sequence (TR = 3,000 ms, α = 25°, 256 averages) before and up to 1.5 h after BP administration. In this way we achieved the spatial localization within the tumor by the shape and dimension of the surface coil (which enables the tumor to be positioned inside it) and the short duration of the flip angle preserving a temporal resolution of 10 minutes.

### Statistical analysis

Differences between treatment groups, both *in vitro* and *in vivo*, were analysed by one-way ANOVA and Bonferroni t-test. Data are expressed as mean ± SD and SE for *in vivo* experiments, and p values reported are two-sided. P values < 0.05 were considered as statistically significant. Statistical analysis was performed with Sigmastat 2006 software.

## Results

### *In vitro* observations of exposure of melanoma cell lines to solubilized commercial alkaline supplement (BP)

**Fig 1:**
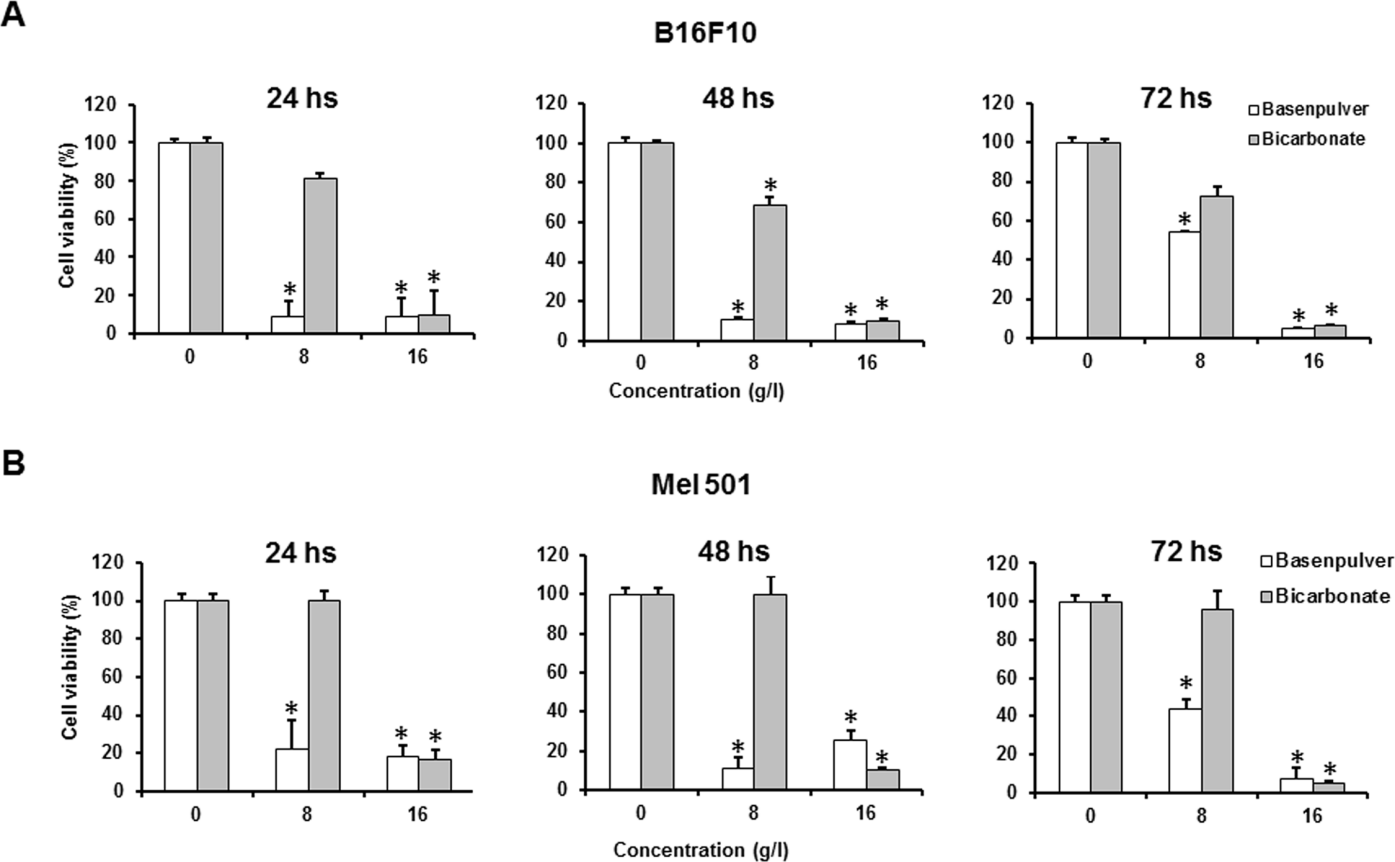
Comparison of the antiproliferative properties of BP versus Sodium Bicarbonate. *In vitro* antiproliferative effect of BP in comparison with sodium bicarbonate against murine melanoma B16F10 (A) and human melanoma Mel 501 (B), A375 (C) at 24, 48 and 72 hours, as indicated. The figures show an increased efficacy of the commercial buffer over sodium bicarbonate (p < 0.05) at 8g/l and at all different setting time. Columns are mean percentages of two independent experiments run in triplicate; bars indicate SD. (*) indicate p < 0.05.

The preliminary experiments were aimed at assessing and standardizing the protocol to promote the best solubility of BP at different concentrations in an acid microenvironment. The high acidity was instrumental to induce the solubility of the alkaline supplement since the carbonate that is present within the alkalizing mix has a poor water solubility at pH values approaching neutrality. Following the addition of sodium bicarbonate or BP to the acidified RPMI the pH increased up to a value of 7.0. These tests showed the BP could be successfully solubilized up to concentrations of 16 g/l at the pH of 1.0 (that is the pH measured in the stomach during the physiological process of digestion). Secondarily, we wished to evaluate if BP at the concentration of 8 and 16 g/l could affect the viability of different melanoma cell lines (Mel 501, B16F10, A375, Me30966 and WM793). In this set of experiments the alkalized culture medium was added to the melanoma cells. The experiments, summarized in Fig 1 for Mel 501 and B16F10 and in S1 Fig for A375, Me30966 and WM793 cell lines, showed that at all setting times (i.e. 24, 48 and 72 hours), BP-dependent alkalization resulted in decreased viability in both cell lines compared to control (p < 0.05) at the concentration of 8 g/l, while the concentration of 16 g/l was exceedingly toxic. Similarly, sodium bicarbonate was administered at the same concentrations to the melanoma cells. However, sodium bicarbonate showed a variable inhibitory activity, from totally absent to barely detectable, against all analyzed melanoma cell lines (Fig 1 and S1 Fig). Moreover, comparably to BP, bicarbonate showed toxic effects at the highest concentration (16 g/l). The analysis of the results of this set of experiments showed that BP induced a significantly higher inhibition of proliferation as compared to both untreated controls and sodium bicarbonate, in all the human and murine melanoma cell lines tested in this study (p < 0.05) (Fig 1 and S1 Fig).

### Alkaline supplementation results in *in vivo* delayed tumor growth

**Fig 2:**
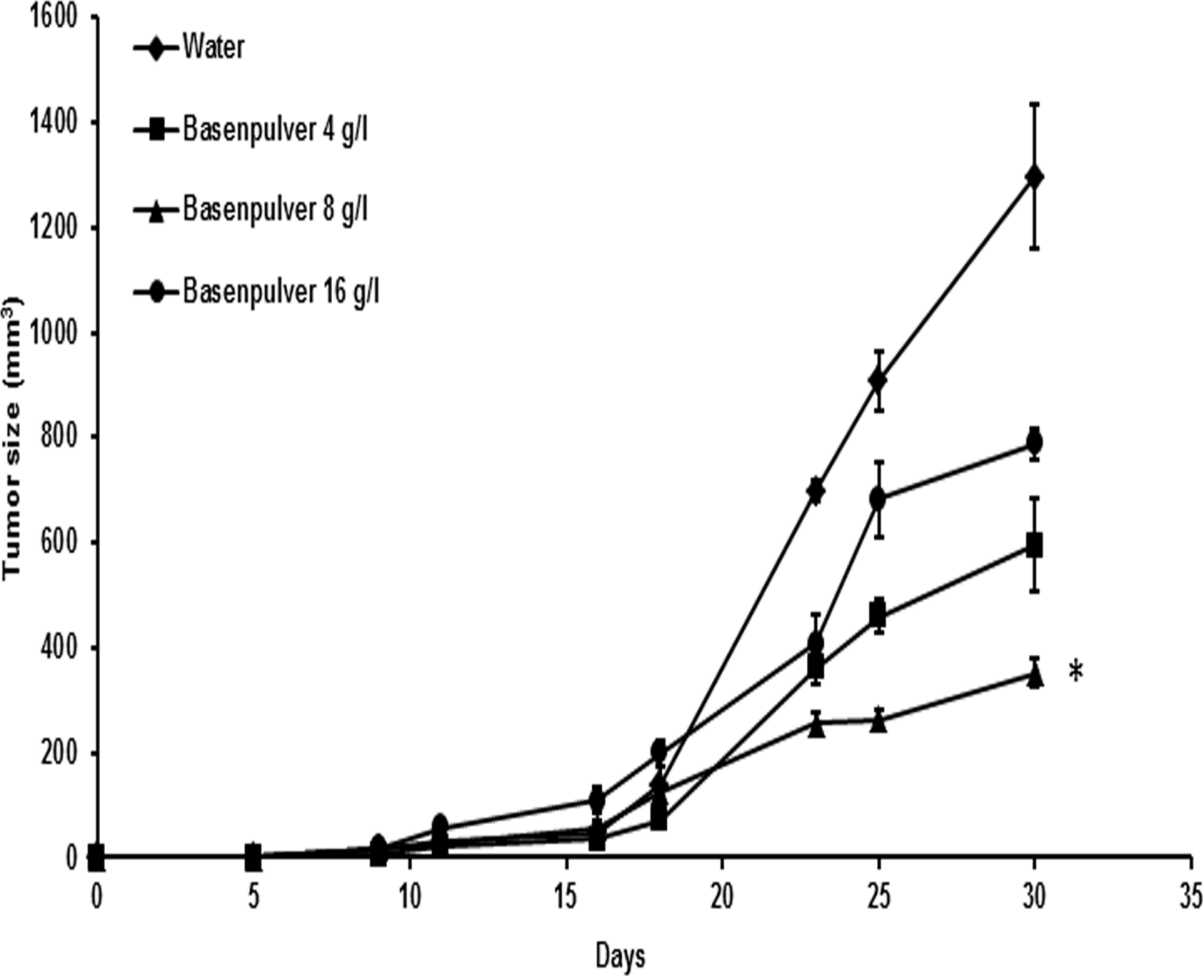
*In vivo* tumor growth inhibition induced by BP. BP shows a dose dependent inhibition of tumor growth in the melanoma B16F10/syngenic mouse model of cutaneous melanoma compared to untreated controls. Tumor inhibition peaked at the BP concentration of 8 g/l, it was less at the concentration of 4 g/l and had a paradoxical decrease at the highest concentration of 16 g/l (p < 0.05). Bars indicate SE. (*) indicate p < 0.05.

In the singeneic mouse model, all the mice, engrafted with B16F10 melanoma cell line, receiving alkaline supplementation by oral gavage as a daily single dose showed delayed tumor growth compared to controls (Fig 2). The 8 g/l was the most effective dose in terms of tumor growth control compared to 4 and 16 g/l. The mice treated with 8 g/l did not show systemic signs of illness (weight loss, poor or unkempt hair coat, decreased activity etc., data not shown). On the contrary, mice receiving the highest dose (16 g/l) had a lesser tumor response and, more importantly, showed side effects due to the therapy. Weight loss, poor hair coat with alopecia confined to the neck, decreased activity and non-cancer-related early death were observed in this last group. In terms of outcome, the group receiving the highest dose of alkalizer had a paradoxical response with a lower tumor growth delay compared to both other treatment groups. In summary, the group receiving the dose of 8 g/l had the best response in terms of tumor growth and overall animal welfare. The growth inhibition experiment lasted 30 days, at which time all the mice in the control group, having reached the maximum tumor volume (1200 mm^3^) allowed by the current law regulating laboratory animals welfare, were sacrificed. The treatment groups had much lower tumor volumes, as shown in Fig 2.

Metastases were not been detected in any mice of the 4 experimental groups. In the mice receiving the alkalizing agent, special care was exerted to check the gastrointestinal mucosa for signs of atrophy or other pathologies potentially related to the treatment. None of the animals had any lesions that could be attributed to the alkalizing therapy.

### Alkaline supplementation results in alkalization of the tumors as well as the urine of the experimental mice

**Fig 3:**
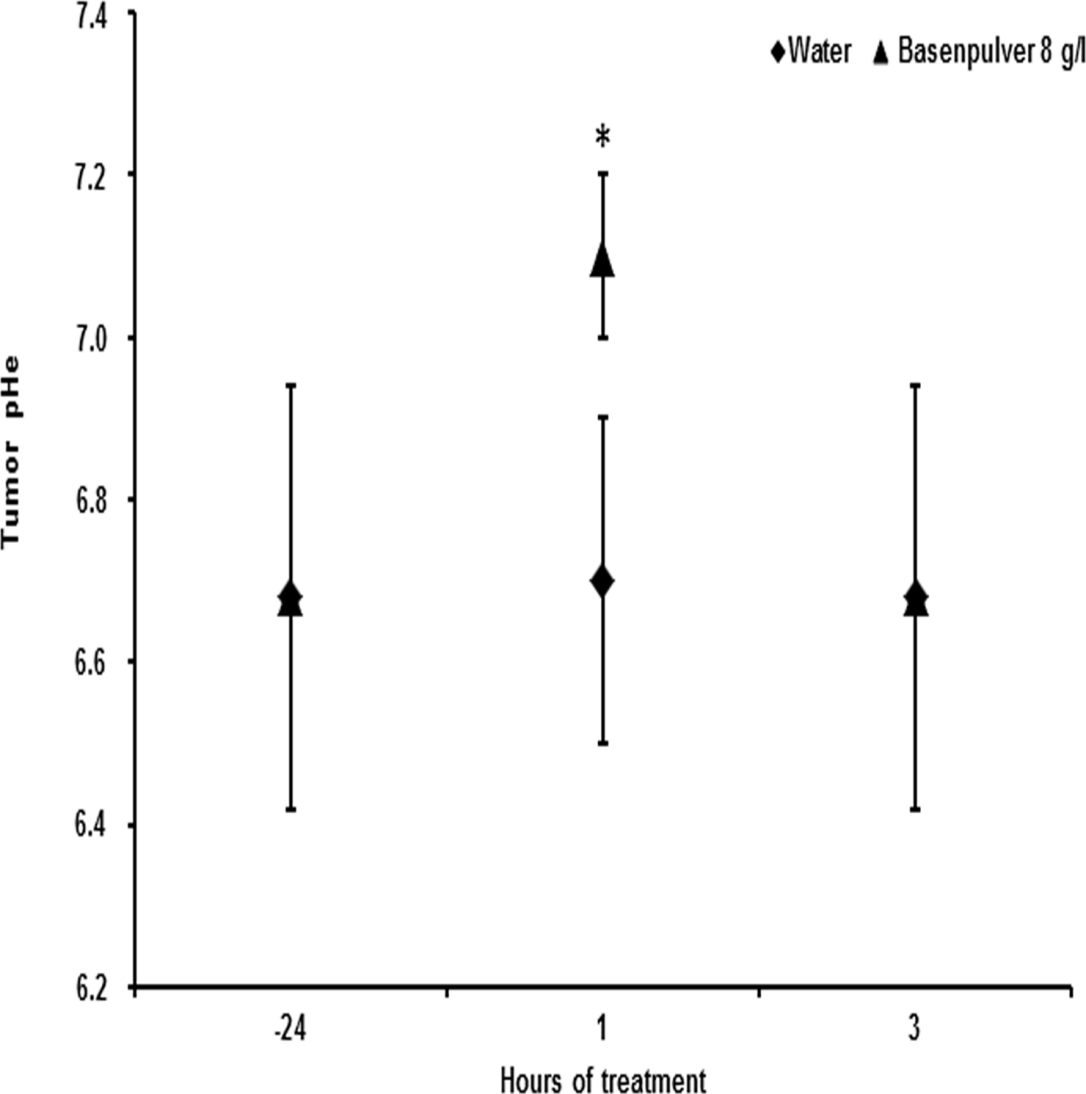
Tumor alkalization induced by single dose of BP detected by ^31^P MRS. Effects of BP administration on tumor extracellular pH measured by *in vivo* ^31^P MRS. Tumors had an average extracellular pH (pHe) of 6.68 ± 0.26 at 24 h before treatment and 7.1 ± 0.1 at 1 hour after treatment (8 g/l). Bars indicate SD. (*) indicate p < 0.05.

This set of experiments was aimed at establishing a correlation between the tumor growth delay and alkalization at both tumor and systemic levels. To this purpose we performed *in vivo* ^31^P magnetic resonance spectroscopy (MRS) of the mice under general anesthesia, as previously described in either xenograft [18] or syngenic [41] tumor models. The MRI/MRS study was performed before and after the daily administration of alkalizer by gavage. Measures were taken every 10 minutes, from 30 to 90 minutes after gavage. The measurements were confined to controls and mice receiving the most tolerable doses of 4 and 8 g/l. Untreated mice had a tumor extracellular pH (expressed as a mean value) of 6.68 ± 0.26 (Fig 3).

**Fig 4:**
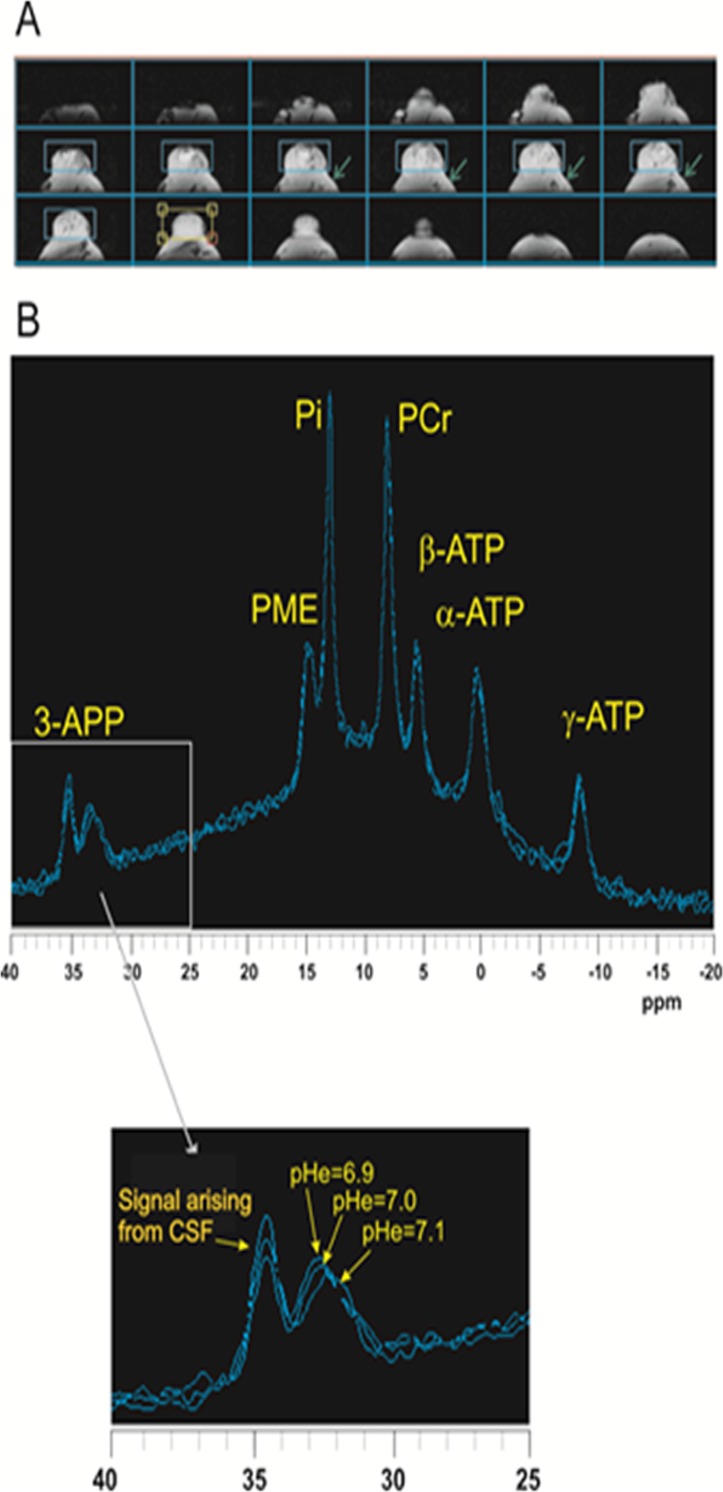
*In vivo* 31P MR spectra acquired at different time after BP administration. A) Axial T1-weighted gradient-echo multislice images from a typical B16F10 tumor 20 days after implant. The light blu rectangles indicate the region of magnetic field optimization. B) *In vivo* ^31^P spectra acquired at different time after Basenpulver 8 g/L administration: from 40 to 50 minutes (pHe = 7.1), from 50 to 60 minutes (pHe = 7.0) and from 60 to 70 minutes (pHe = 6.9). A small amount of contamination arising from the most acid 3-APP signal (at 34 ppm) and from PCr could be due to CSF and muscle, respectively, because of the close proximity of the implanted tumor to the vertebral column (green arrows). However, the small PCr/α-ATP ratio and the presence of the PME signal ensure the tumor origin of the spectrum. Assignments: 3-APP, 3-amino-propil-phosphonate; PME, phosphomonoesters; Pi, inorganic phosphate; PDE, phosphodiesters; ATP, adenosine triphosphate.

In the two treatment groups, maximum tumor alkalization was reached after 1 hour from the administration of the alkalizer agent. *In vivo* ^31^P MR spectra acquired at different time after BP administration are show in Fig 4. Mice receiving 4 g/l of BP had a change in the extracellular pH that was not statistically significant compared to control group (data not show). On the other hand, in the treatment group receiving the dose of 8 g/l, the extracellular pH became 7.1 ± 0.1.

**Fig 5:**
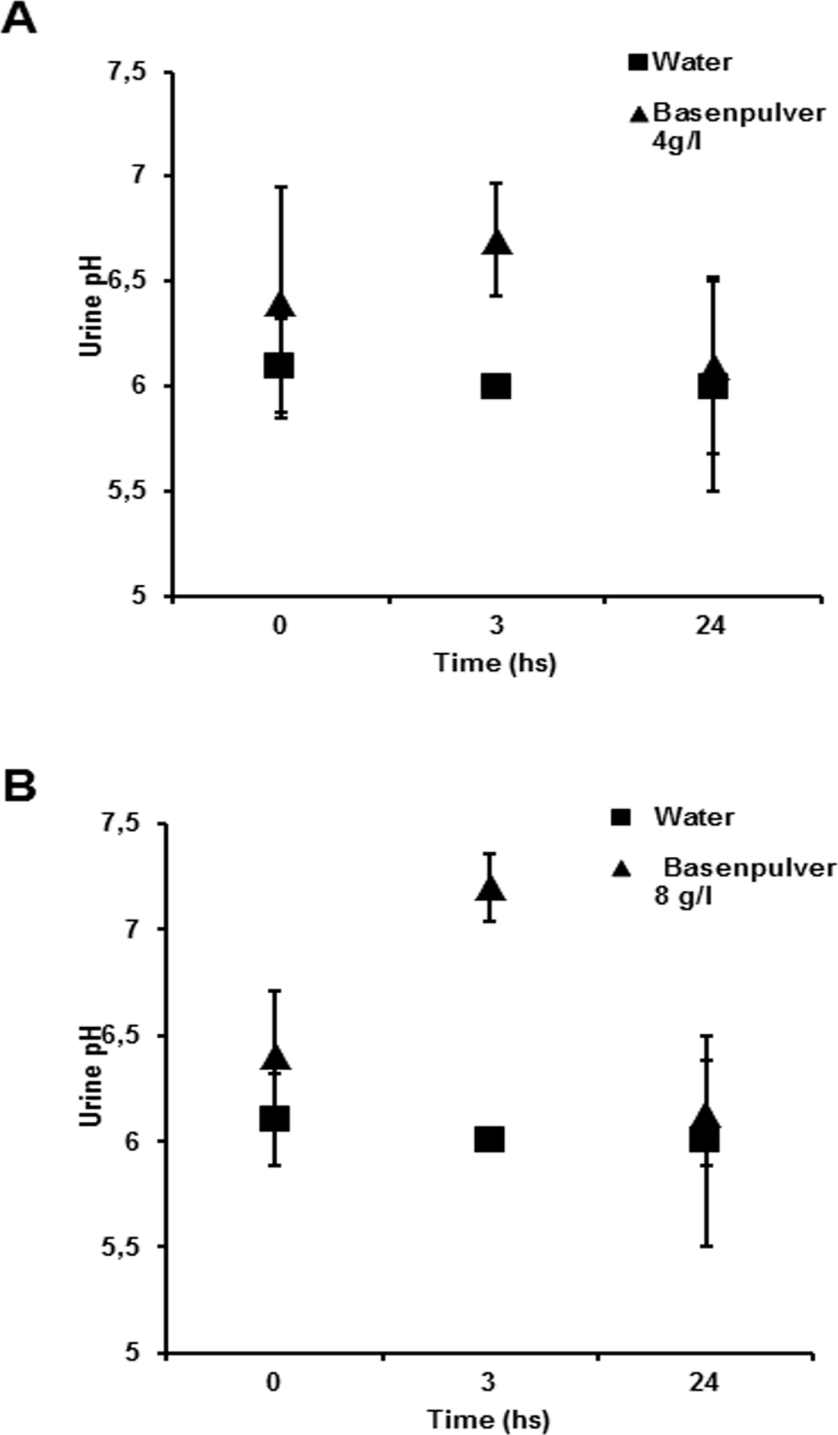
Urine alkalization following the administration of single dose of BP. Effects of BP administration on urine pH (measured with urine strips) at 0, 3 and 24 hours at the BP therapeutic concentrations of 4 and 8 g/l. The urine alkalization reached the zenith at 3 hours and then normalized over time; the peak values were 6.7 ± 0.26 and 7.2 ± 0.15 for the 4 g/l (A) and 8 g/l (B) concentrations respectively. Bars indicate SD. (*) indicate p < 0.05.

Together with measuring changes in tumor pH, we analyzed the effect of BP on systemic pH by measuring the pH of the urines of treated animals. The results showed that BP induced a time-dependent alkalinization of the mice urines in a dose-dependent manner. Briefly, the untreated control mice had a urine pH of 6.0, while in mice treated with BP at the concentration of 4 g/l the urine pH reached values of 6.6 ± 0.26 after 3 hours from the BP oral treatment (Fig 5A). The urine pH values of the treated animals reversed back to values comparable to those of untreated animals within the 24 hours following the BP treatment. Mice receiving BP at the concentration of 8 g/l, reached even higher values of urine pH (7.1 ± 0.1) with the same recovery during the following 24 hours (Fig 5B).

All in all this set of results showed that the effect of BP treatment on tumor growth was consistent with an increase of pH at both tumor and systemic (urine) levels, but exclusively with the dose of 8 g/l.

## Discussion

Tumor acidity is a known feature that is getting more and more attention from basic researchers to clinicians as well [3]. It has been shown that the extracellular release of H^+^ by proton transporters is responsible for the counter gradient observed in tumor cells compared to normal cells [23]. These transporters include V-ATPases, NHE, and lactate/H^+^ symporter, and are responsible, along with the participation of carbonic anhydrase, for the continuous release of proton excess secondary to the anaerobic metabolism of glucose. This results in an intracellular alkaline pH and an extracellular acid pH that leads, among the other effects, to lymphocyte anergy and activation of the proteolytic enzymes responsible for local invasiveness and metastases. Moreover this complex phenomenon is responsible for the chemoresistance of tumors, in particular solid variants [9]. Several strategies have been adopted to overcome this property of cancer cells that is now being considered a phenotype per se [5,8]. Our group and others have been working on the inhibition of one or more proton transporters, with remarkable results in preclinical models and also in clinical trials [26–28,41]. We have also been considering a more thorough approach through the alkalization of the patient as a whole to overcome tumor acidity [28]. In this study we evaluated the contribution of water alkalization to the efficacy of metronomic chemotherapy in pet patients receiving high doses of proton pump inhibitors, obtaining high percentages of response [28]. A pioneer investigation has evaluated the impact of sodium bicarbonate administration in the water on the development of prostate carcinoma in TRAMP mice, obtaining the prevention of this cancer by administering buffered water since an early age [31]. In the current study we wished to quantify and characterize the tumor control potential of alkaline buffer on the tumor growth and progression in C57/BL mice carrying B16F10 syngenic melanoma, that is a very aggressive tumor. Moreover, we wished to evaluate if it was possible to modulate tumor acidic microenvironment with single daily doses of alkali rather than by continuous administration with the water, to overcome a practical issue that has been raised by the first attempts to translate bicarbonate therapy in clinic: human patients, differently from mice that crave the substance, have a low tolerance for water highly saturated with sodium bicarbonate. Our investigation showed that it was possible to successfully alkalize tumor microenvironment using a commercially available alkaline supplementation (Basenpulver®, BP) without causing undesirable toxicosis in the laboratory animals. This preparation offers several advantages: 1) it is a mix of carbonates and bicarbonates; 2) it has a cation composition that is more similar to the one that is physiologically found in healthy individuals. In fact one of the possible clinical drawbacks of bicarbonate is the high sodium content of the product that, when consumed in high doses, might lead, over time, to water retention and cardiac impairment. In terms of biological advantages provided by the adoption of a balanced mix of carbonate and bicarbonates that make up BP, the following should be noted: a) when the bicarbonate component is spent by neutralizing tumor derived protons, being converted in water and carbon dioxide, the carbonate becomes bicarbonate accordingly to the dissociation constant of the molecule; b) the mix of alkali, differently from what happens *in vitro*, interact also with the physiological buffering systems of the individual; c) while *in vivo* the bicarbonates are partially or completely consumed by the gastric acidity, the carbonates are able to bypass the stomach and remain available for systemic consumption after their transformation in bicarbonates. BP showed a very good inhibitory activity at the dose of 8 g/l, through an alkalization mechanism evidenced by MRI as well as urine analysis. The dose of 16 g/l has proven to be still effective (albeit less effective than the 8 g/l dose) but detrimental in terms of general conditions of the mice. These undesired complications could probably account for its reduced effectiveness. The lowest dose of 4 g/l has been included to monitor for side effects at suboptimal dose. This dose was well-tolerated, induced urine alkalization but failed to alkalize the tumor periphery. However a residual efficacy was still present as shown by the delayed tumor growth in mice receiving this dose. The exact mechanism of this phenomenon still eludes us, however we can hypothesize that even at the lower dose, BP still added to the individual buffering resources modulating tumor microenvironment. This unexpected finding suggests that alkalizing therapy could still be considered for individuals unable to tolerate the optimal dose (8 g/l) due to underlying gastrointestinal and systemic conditions. However, it is conceivable that the *in vivo* treatment partially stimulated the immune response, due to the tumor microenvironment alkalinization. In fact, in a previous study we showed that acidity may represent a tumor immune escape mechanism per se and that treatment with a family of very potent anti-acidic molecules, such as proton pump inhibitors, inhibited the tumor growth with a clear increase of both adoptive and local tumor immune infiltrate in a mouse model very similar to the one used in this study [42]. However, in this study we have also shown that reproducing *in vitro* a gastric pH-like BP activation, we had a direct antitumor effect of the alkalinizer on tumor cells, suggesting that potent alkalinization impairs tumor cell viability per se.

## Conclusion

In summary, water alkalization provides an inexpensive, highly effective and overall well-tolerated means to counter one of the most malignant feature of tumors. As part of a prevention protocol, it might help to protect individuals at risk from developing aggressive malignancies. Moreover, acidity being a very efficient mechanism of tumor resistance to drugs [8]. It is conceivable that controlling pH imbalances at the level of organs and compartments that are the target of current anti-cancer therapies may represent an efficient new tool for improving drug efficacy and that these results will promote further investigations into combining different families of alkalizers with standard chemotherapy and immunomodulating agents.

We want to conclude with an emphasis to the fact that the achievement of these results, with a high potential to be translated to the clinical use, was obtained thanks to the nonmainstream approach against cancer, representing a key paradigm of our research group [43].

## Supporting Information

S1 Fig Comparison of the antiproliferative properties of BP versus Sodium Bicarbonate. *In vitro* antiproliferative effect of BP in comparison with sodium bicarbonate against human melanoma A375 (A), Me30966 (B) and WM794 (C) at 24, 48, 72 hours, as indicated. The figures show an increased efficacy of the commercial buffer over sodium bicarbonate (p < 0.05) at 8g/l and at all different setting time. The experiments performed at 96 hours did not show significant differences to the inhibition obtained at the earliest time points. Columns are mean percentages of two independent experiments run in triplicate; bars indicate SD. (*) indicate p < 0.05.
(PDF)

## References

[1] Gatenby RA, Gillies RJ. Why do cancers have high aerobic glycolysis? Nature Rev Cancer. 2004;4: 891–899. 15516961 10.1038/nrc1478

[2] De Milito A and Fais S. Tumor acidity, chemoresistance and proton pump inhibitors. Future Oncol. 2005;1: 779–786. 16556057 10.2217/14796694.1.6.779

[3] Fais S, Venturi G, Gatenby B. Microenvironmental acidosis in carcinogenesis and metastases: new strategies in prevention and therapy. Cancer Met Rev. 2014; 33: 1095–108. 10.1007/s10555-014-9531-3 PMC4244550 25376898

[4] Cairns RA, Harris IS, Mak TW. Regulation of cancer cell metabolism, Nat. Rev. Cancer. 2011;11: 85–95. 10.1038/nrc2981 21258394

[5] Raz S, Sheban D, Gonen N, Stark M, Berman B, Assaraf YG. Severe hypox induces complete antifolate resistance in carcinoma cells due to cell cycle arrest. Cell Death Dis. 2014;5: e1067 10.1038/cddis.2014.39 24556682 PMC3944254

[6] Spugnini EP, Sonveaux P, Stock C, Perez-Sayans M, De Milito A, Avnet S, et al Proton channels and exchangers in cancer. Biochim Biophys Acta. 2015;10 Pt B: 2715–26. 10.1016/j.bbamem.2014.10.015 25449995

[7] Bellone M, Calcinotto A, Filipazzi P, De Milito A, Fais S, Rivoltini L. The acidity of the tumor microenvironment is a mechanism of immune escape that can be overcome by proton pump inhibitors. Oncoimmunology. 2013;2: e22058 23483769 10.4161/onci.22058 PMC3583905

[8] Taylor S, Spugnini EP, Assaraf YG, Azzarito T, Rauch C, Fais S. Microenvironment acidity as a major determinant of tumor chemoresistance: Proton pump inhibitors (PPIs) as a novel therapeutic approach, Drug Resist Updat. 2015;23:69–78. 10.1016/j.drup.2015.08.004 26341193

[9] Raghunand N, Mahoney BP, Gillies RJ. Tumor acidity, ion trapping and chemotherapeutics. II. pH-dependent partition coefficients predict importance of ion trapping on pharmacokinetics of weakly basic chemotherapeutic agents. Biochem Pharmacol. 2003;66: 1219–29. 14505801 10.1016/s0006-2952(03)00468-4

[10] Parks SK, Chiche J, Pouysségur J. Disrupting proton dynamics and energy metabolism for cancer therapy. Nat Rev Cancer. 2013;13: 611–23. 10.1038/nrc3579 23969692

[11] Huber V, De Milito A, Harguindey S, Reshkin SJ, Wahl ML, Rauch C, et al Proton dynamics in cancer. J Transl Med. 2010;8: 57 10.1186/1479-5876-8-57 20550689 PMC2905351

[12] Estrella V, Chen T, Lloyd M, Wojtkowiak J, Cornnell HH, Ibrahim-Hashim A, et al Acidity generated by the tumor microenvironment drives local invasion. Cancer Res. 2013;73:1524–35. 10.1158/0008-5472.CAN-12-2796 23288510 PMC3594450

[13] Fais S. Proton pump inhibitor-induced tumour cell death by inhibition of a detoxification mechanism. J. Intern. Med. 2010;267: 515–525. 10.1111/j.1365-2796.2010.02225.x 20433578

[14] De Milito, Marino ML, and Fais S. A rationale for the use of proton pump inhibitors as antineoplastic agents. Curr. Pharm. Des. 2012;18: 1395–1406. 22360553 10.2174/138161212799504911

[15] Olbe L, Carlsson E, and Lindberg P. A proton-pump inhibitor expedition: The case histories of omeprazole and esomeprazole. Nat. Rev. Drug Discov. 2003;2: 132–139. 12563304 10.1038/nrd1010

[16] Luciani F, Spada M, De Milito A, Molinari A, Rivoltini L, Montinaro A, et al Effect of proton pump inhibitor pretreatment on resistance of solid tumors to cytotoxic drugs. J. Natl. Cancer Inst. 2004;96: 1702–1713. 15547183 10.1093/jnci/djh305

[17] De Milito A, Iessi E, Logozzi M, Lozupone F, Spada M, Marino ML, et al Proton pump inhibitors induce apoptosis of human B-cell tumors through a caspase-independent mechanism involving reactive oxygen species, Cancer Res. 2007;67: 5408–5417. 17545622 10.1158/0008-5472.CAN-06-4095

[18] De Milito A, Canese R, Marino ML, Borghi M, Iero M, Villa A, et al pH-dependent antitumor activity of proton pump inhibitors against human melanoma is mediated by inhibition of tumor acidity. Int. J. Cancer. 2010;127: 207–219. 10.1002/ijc.25009 19876915

[19] Marino ML, Fais S, Djavaheri-Mergny M, Villa A, Meschini S, Lozupone F, et al Proton pump inhibition induces autophagy as a survival mechanism following oxidative stress in human melanoma cells. Cell. Death Dis. 2010;1: e87 10.1038/cddis.2010.67 21368860 PMC3035900

[20] Udelnow A, Kreyes A, Ellinger S, Landfester K, Walther P, Klapperstueck T, et al Omeprazole inhibits proliferation and modulates autophagy in pancreatic cancer cells. PLoS One. 2011;6: e20143 10.1371/journal.pone.0020143 21629657 PMC3101238

[21] Chen M, Zou X, Luo H, Cao J, Zhang X, Zhang B, et al Effects and mechanisms of proton pump inhibitors as a novel chemosensitizer on human gastric adenocarcinoma (SGC7901) cells. Cell Biol. Int. 2009;33: 1008–1019. 10.1016/j.cellbi.2009.05.004 19501661

[22] Chen m, Huang SL, Zhang XQ, Zhang B, Zhu H, Yang VW, et al Reversal effects of pantoprazole on multidrug resistance in human gastric adenocarcinoma cells by down-regulating the V-ATPases/mTOR/HIF-1alpha/P-gp and MRP1 signaling pathway in vitro and in vivo. J. Cell. Biochem. 2012;113: 2474–2487. 10.1002/jcb.24122 22396185 PMC3762681

[23] Lugini L, Federici C, Borghi M, Azzarito T, Marino ML, Cesolini A, et al Proton pump inhibitors while belonging to the same family of generic drugs show different anti-tumor effect, J Enzyme Inhib Med Chem. 2015;28: 1–8. 10.3109/14756366.2015.1046062 26018420

[24] Azzarito T, Venturi G, Cesolini A, Fais S. Lansoprazole induces sensitivity to suboptimal doses of paclitaxel in human melanoma. Cancer Lett. 2015;356(2 Pt B): 697–703. 10.1016/j.canlet.2014.10.017 25449440

[25] Walsh M, Fais S, Spugnini EP, Harguindey S, Abu Izneid T, Scacco L, et al Proton pump inhibitors for the treatment of cancer in companion animals, J Exp Clin Cancer Res. 2015;34: 93 10.1186/s13046-015-0204-z 26337905 PMC4559889

[26] Ferrari S, Perut F, Fagioli F, Brach Del Prever A, Meazza C, et al Proton pump inhibitor chemosensitization in human osteosarcoma: from the bench to the patients' bed. J Transl Med. 2013;11: 268 10.1186/1479-5876-11-268 24156349 PMC3815282

[27] Wang BY, Zhang J, Wang JL, Sun S, Wang ZH, Wang LP, et al Intermittent high dose proton pump inhibitor enhances the antitumor effects of chemotherapy in metastatic breast cancer. J Exp Clin Cancer Res. 2015;34: 85 10.1186/s13046-015-0194-x 26297142 PMC4546346

[28] Spugnini EP, Buglion S, Carocci F, Francesco M, Vincenzi B, Fanciulli M, et al High dose lansoprazole combined with metronomic chemotherapy: a phase I/II study in companion animals with spontaneously occurring tumors, J Transl Med. 2014;12: 225 10.1186/s12967-014-0225-y 25143012 PMC4145230

[29] Ribeiro MD, Silva AS, Bailey KM, Kumar NB, Sellers TA, Gatenby RA, et al Buffer Therapy for Cancer. J Nutr Food Sci. 2012;2: 6 24371544 PMC3872072

[30] Silva SA, Yunes JA, Gillies RJ, Gatenby RA. The potential role of systemic buffers in reducing intratumoral extracellular pH and acid-mediated invasion. Cancer Res. 2009;69: 2677–84. 10.1158/0008-5472.CAN-08-2394 19276380 PMC3718046

[31] Ibrahim-Hashim A, Cornnell HH, Abrahams D, Lloyd M, Bui M, Gillies RJ, et al Systemic buffers inhibit carcinogenesis in TRAMP mice. J Urol. 2012;188: 624–31. 10.1016/j.juro.2012.03.113 22704445 PMC3694604

[32] Jackson SL, King SM, Zhao L, Cogswell ME. Prevalence of Excess Sodium Intake in the United States-NHANES, 2009–2012. MMWR Morb Mortal Wkly Rep. 2016; 64: 1393–7. 10.15585/mmwr.mm6452a1 26741238

[33] DiNicolantonio JJ, Chatterjee S, O'Keefe JH. Dietary Salt restriction in Heart Failure: Where is the Evidence? Prog Cardiovasc Dis. 2015;15 pii: S0033-0620. 10.1016/j.pcad.2015.12.002 26721179

[34] Suckling RJ, Swift PA. The health impacts of dietary sodium and a low-salt diet. Clin Med (Lond). 2015;15: 585–8. 26621954 10.7861/clinmedicine.15-6-585 PMC4953267

[35] Blumenthal JA, Sherwood A, Smith PJ, Mabe S, Watkins L, Lin PH, et al Lifestyle modification for resistant hypertension: The TRIUMPH randomized clinical trial. Am Heart J. 2015;170: 986–994. 10.1016/j.ahj.2015.08.006 26542509 PMC4636732

[36] Ota T, Uchida S. [Kidney diseases and metabolic disorders-Basics and applications required for general physicians. Topics: I. Disorders of water balance and sodium metabolism]. Nihon Naika Gakkai Zasshi. 2015;104: 906–16. 26591339 10.2169/naika.104.906

[37] Harring TR, Deal NS, Kuo DC. Disorders of sodium and water balance. Emerg Med Clin North Am. 2014;2: 379–401. 10.1016/j.emc.2014.01.001 24766939

[38] Tomey MI, Winston JA. Cardiovascular pathophysiology in chronic kidney disease: opportunities to transition from disease to health. Ann Glob Health. 2014;80: 69–76. 10.1016/j.aogh.2013.12.007 24751567

[39] Morton DB and Griffiths PH. Guidelines on the recognition of pain, distress and discomfort in experimental animals and an hypothesis for assessment. Vet. Rec. 1985;116: 431–436. 3923690 10.1136/vr.116.16.431

[40] Tremoleda J.L., Kerton A., Gsell W. Anaesthesia and physiological monitoring during in vivo imaging of laboratory rodents: considerations on experimental outcomes and animal welfare, EJNMMI Res. 2012;2: 44 10.1186/2191-219X-2-44 22877315 PMC3467189

[41] Spugnini EP, Baldi A, Buglioni S, Carocci F, de Bazzichini GM, Betti G, et al Lansoprazole as a rescue agent in chemoresistant tumors: a phase I/II study in companion animals with spontaneously occurring tumors, J Transl Med. 2011;9: 221 10.1186/1479-5876-9-221 22204495 PMC3264547

[42] Calcinotto A, Filipazzi P, Grioni M, Iero M, De Milito A, Ricupito A, et al Modulation of microenvironment acidity reverses anergy in human and murine tumor-infiltrating T lymphocytes. Cancer Res. 2012;72: 2746–56. 10.1158/0008-5472.CAN-11-1272 22593198

[43] Fais S. A nonmainstream approach against cancer. J Enzyme Inhib Med Chem. 2016 3 14:1–8. [Epub ahead of print] 26972280 10.3109/14756366.2016.1156105

