# Supplementary figures and images for: Effect of Modified Alkaline Supplementation on Syngenic Melanoma Growth in CB57/BL Mice
Source: PLoS One. 2016 Jul 22;11(7):e0159763. doi: 10.1371/journal.pone.0159763 (PMC4957829; doi:10.1371/journal.pone.0159763)

**A**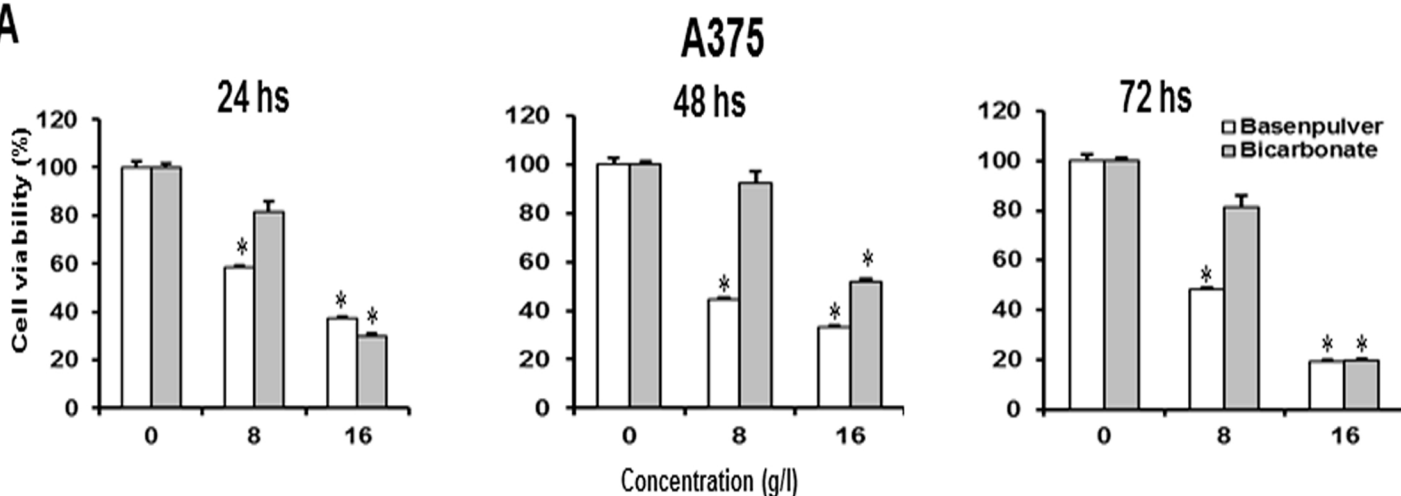**B**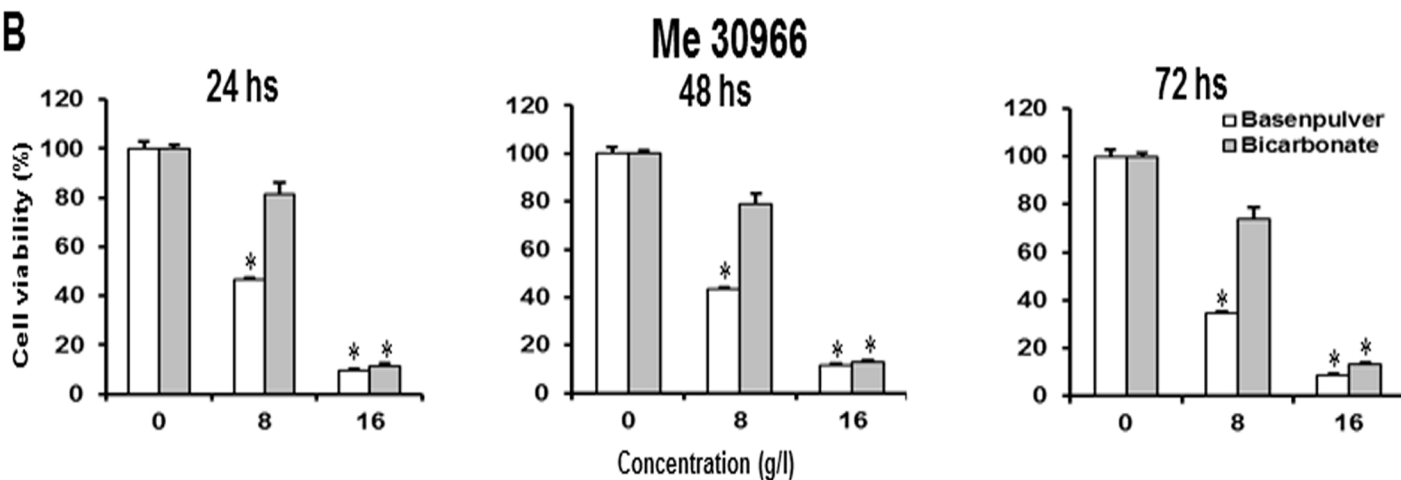**C**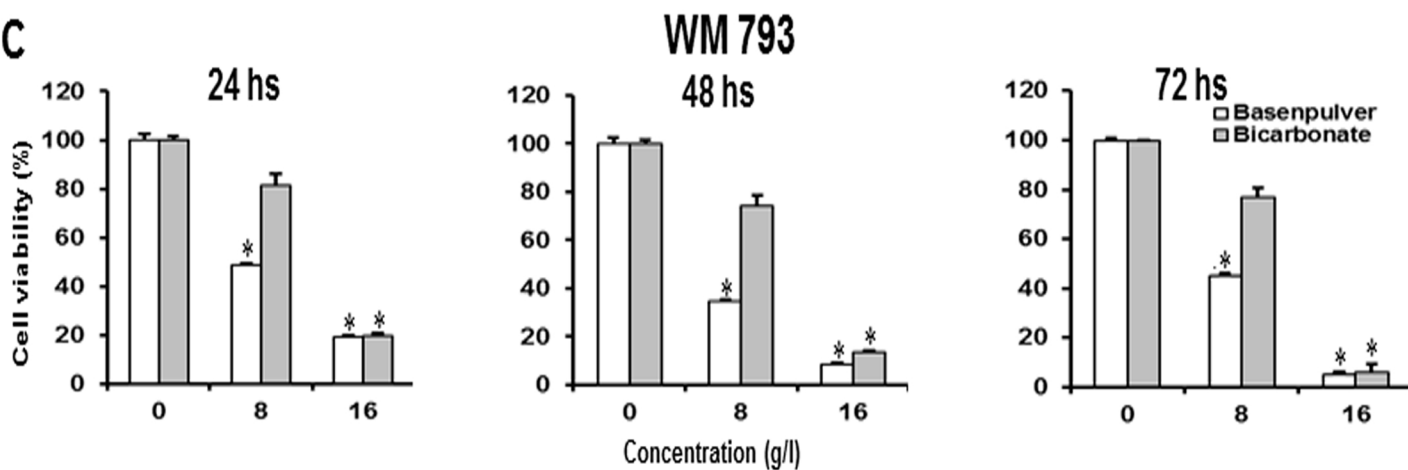**S 1**

Supplement: S1 Fig — In vitro antiproliferative effect of BP in comparison with sodium bicarbonate against human melanoma A375 (A), Me30966 (B) and WM794 (C) at 24, 48, 72 hours, as indicated. The figures show an increased efficacy of the commercial buffer over sodium bicarbonate (p < 0.05) at 8g/l and at all different setting time. The experiments performed at 96 hours did not show significant differences to the inhibition obtained at the earliest time points. Columns are mean percentages of two independent experiments run in triplicate; bars indicate SD. (*) indicate p < 0.05. (PDF) [file pone.0159763.s001.pdf]
